# Predictive value of neutrophil-to-lymphocyte ratio for the fatality of COVID-19 patients complicated with cardiovascular diseases and/or risk factors

**DOI:** 10.1038/s41598-022-17567-4

**Published:** 2022-08-10

**Authors:** Akinori Higaki, Hideki Okayama, Yoshito Homma, Takahide Sano, Takeshi Kitai, Taishi Yonetsu, Sho Torii, Shun Kohsaka, Shunsuke Kuroda, Koichi Node, Yuya Matsue, Shingo Matsumoto

**Affiliations:** 1 Department of Cardiology, Ehime Prefectural Central Hospital, Matsuyama, Japan; 2 Department of Infectious Diseases, Ehime Prefectural Central Hospital, Matsuyama, Japan; 3 Department of Cardiovascular Medicine, Toho University Graduate School of Medicine, Tokyo, Japan; 4 Department of Cardiovascular Medicine, National Cerebral and Cardiovascular Center, Osaka, Japan; 5 Department of Rehabilitation, Kobe City Medical Center General Hospital, Kobe, Japan; 6 Department of Interventional Cardiology, Tokyo Medical and Dental University, Tokyo, Japan; 7 Department of Cardiology, Tokai University School of Medicine, Isehara, Japan; 8 Department of Cardiology, Keio University School of Medicine, Tokyo, Japan; 9 Department of Cardiovascular Medicine, Cleveland Clinic, Cleveland, Ohio USA; 10 Department of Cardiovascular Medicine, Saga University, Saga, Japan; 11 Department of Cardiovascular Biology and Medicine, Juntendo University Graduate School of Medicine, Tokyo, Japan; 12 Cardiovascular Respiratory Sleep Medicine, Juntendo University Graduate School of Medicine, Tokyo, Japan; 13 Division of Cardiovascular Medicine, Department of Internal Medicine, Toho University Faculty of Medicine, Tokyo, Japan

**Keywords:** Cardiovascular diseases, Risk factors, Viral infection

## Abstract

Previous studies have reported that a high neutrophil-to-lymphocyte ratio (NLR) is associated with disease severity and poor prognosis in COVID-19 patients. We aimed to investigate the clinical implications of NLR in patients with COVID-19 complicated with cardiovascular diseases and/or its risk factors (CVDRF). In total, 601 patients with known NLR values were selected from the CLAVIS-COVID registry for analysis. Patients were categorized into quartiles (Q1, Q2, Q3, and Q4) according to baseline NLR values, and demographic and clinical parameters were compared between the groups. Survival analysis was conducted using the Kaplan–Meier method. The diagnostic performance of the baseline and follow-up NLR values was tested using receiver operating characteristic (ROC) curve analysis. Finally, two-dimensional mapping of patient characteristics was conducted using t-stochastic neighborhood embedding (t-SNE). In-hospital mortality significantly increased with an increase in the baseline NLR quartile (Q1 6.3%, Q2 11.0%, Q3 20.5%; and Q4, 26.6%; p < 0.001). The cumulative mortality increased as the quartile of the baseline NLR increased. The paired log-rank test revealed significant differences in survival for Q1 vs. Q3 (p = 0.017), Q1 vs. Q4 (p < 0.001), Q2 vs. Q3 (p = 0.034), and Q2 vs. Q4 (p < 0.001). However, baseline NLR was not identified as an independent prognostic factor using a multivariate Cox proportional hazards regression model. The area under the curve for predicting in-hospital death based on baseline NLR was only 0.682, whereas that of follow-up NLR was 0.893. The two-dimensional patient map with t-SNE showed a cluster characterized by high mortality with high NLR at follow-up, but these did not necessarily overlap with the population with high NLR at baseline. NLR may have prognostic implications in hospitalized COVID-19 patients with CVDRF, but its significance depends on the timing of data collection.

## Introduction

As evidence accumulates, it has become clear that the presence of cardiovascular disease (CVD) is closely related to the prognosis of COVID-19 patients^1,2^. Early studies reported that patients who required intensive care were more likely to have CVD^3^, and myocardial injury was associated with fatal outcomes in COVID-19^4^. More recently, Cereda et al. reported that the coronary calcium score contributes to stratifying the risk of complications in COVID-19 patients^5^. Similarly, virus-related cardiac injury has also been highlighted through investigations of hospitalized patients^6^. Viral infections are often associated with lymphopenia, and COVID-19 is no exception to this. Several studies have found a correlation between disease severity and lymphopenia^7–9^. On the other hand, the role of neutrophils in COVID-19 is also attracting increasing attention^10^. A recent study by Parackova et al. demonstrated that neutrophils from COVID-19 patients induced T-cell polarization, leading to reduction in the percentage of Th1 cells^11^. In this context, it is suggested that the balance between neutrophils and lymphocytes reflects the disease activity of COVID-19. The neutrophil-to-lymphocyte ratio (NLR) is a biomarker of systemic inflammatory status that can easily be obtained from differential white blood cell count^12^. NLR, calculated as a simple ratio between the neutrophil and lymphocyte counts, reflects the balance between acute and chronic inflammation and is predictive of mortality, even in the general population^13^. A series of recent studies have shown that NLR is an independent risk factor for critical illness and hospital mortality in COVID-19 patients^14,15^. In 2020, Qin et al. reported that plasma T lymphocyte levels were significantly reduced, while neutrophil levels were augmented in patients with severe COVID-19 compared with those in patients with mild symptoms^7^. Importantly, there were significantly more cases of CVD in patients with severe symptoms. Therefore, we believe that the comorbidity of CVDs should be considered when investigating the clinical significance of NLR in COVID-19 patients. In this study, we sought to investigate the prognostic significance of NLR in patients with COVID-19 complicated with CVD and/or its risk factors (CVDRF).

## Methods

### Study design and population

This was a retrospective analysis conducted using Clinical Outcomes of COVID-19 Infection in Hospitalized Patients with Cardiovascular Diseases and/or Risk Factors (CLAVIS-COVID registry). This study was approved by the Ethics Committee of Ehime Prefectural Central Hospital (no. 02-22), and the study protocol complied with the tenets of the Declaration of Helsinki. The CLAVIS-COVID registry is a retrospective, observational, national, multicenter study that included an adult population with CVDRF hospitalized for COVID-19 in Japan. The main aim of the registry was to evaluate the characteristics and clinical outcomes of hospitalized COVID-19 patients with CVDRF. The study protocol including the opt-out consent method was approved by the review board of each institution, and all patients provided informed consent to participate in the study. This clinical study was registered with the University Hospital Medical Information Network Clinical Trial Registry (UMIN-ID: UMIN000040598; further details accessible at https://upload.umin.ac.jp/cgi-open-bin/ctr_e/ctr_view.cgi?recptno=R000046132) before the first patient was enrolled, in accordance with the International Committee of Medical Journal Editors. Detailed inclusion/exclusion criteria, decision of hospitalization/discharge, and definition of collected data are described in the original article^16^. Briefly, a total of 1518 patients were recruited from 49 hospitals from January 1 to May 31, 2020. Among all participants, 693 were complicated with CVDRF. Cardiovascular risk factors were defined as hypertension, diabetes mellitus, and dyslipidemia. Pre-existing CVD was defined as a history and/or manifestations upon admission for any of the following: heart failure, coronary artery disease, myocardial infarction, peripheral artery disease, valvular heart disease, cardiac arrhythmia, pericarditis, myocarditis, congenital heart disease, pulmonary hypertension, deep vein thrombosis, pulmonary embolism, aortic dissection, aortic aneurysm, cerebral infarction/transient ischemic attack, heart transplantation, and cardiac arrest and the use of cardiac devices (e.g., a pacemaker, implantable cardioverter-defibrillator, cardiac resynchronization therapy device, and left ventricular assist device). COVID-19 was diagnosed based on a positive polymerase chain reaction test of nasal or pharyngeal swab specimens in all patients. All patients admitted to the participating hospital and enrolled in this study were discharged by November 8, 2020, the deadline for data transfer. Clinical data, including symptoms, demographics, medical history, home medications, baseline comorbidities, physical findings, laboratory test results, radiography and chest computed tomography findings, electrocardiography and cardiac echocardiography results, treatment information, and outcomes, were obtained from electronic medical records using data collection forms. All laboratory and imaging data were obtained at the time of admission. We defined the data at “follow-up” as the results of the final blood test performed before hospital discharge, regardless of the form of discharge. According to this definition, baseline and follow-up values will be the same in cases wherein only one blood test was performed during hospitalization.

**Figure 1:**
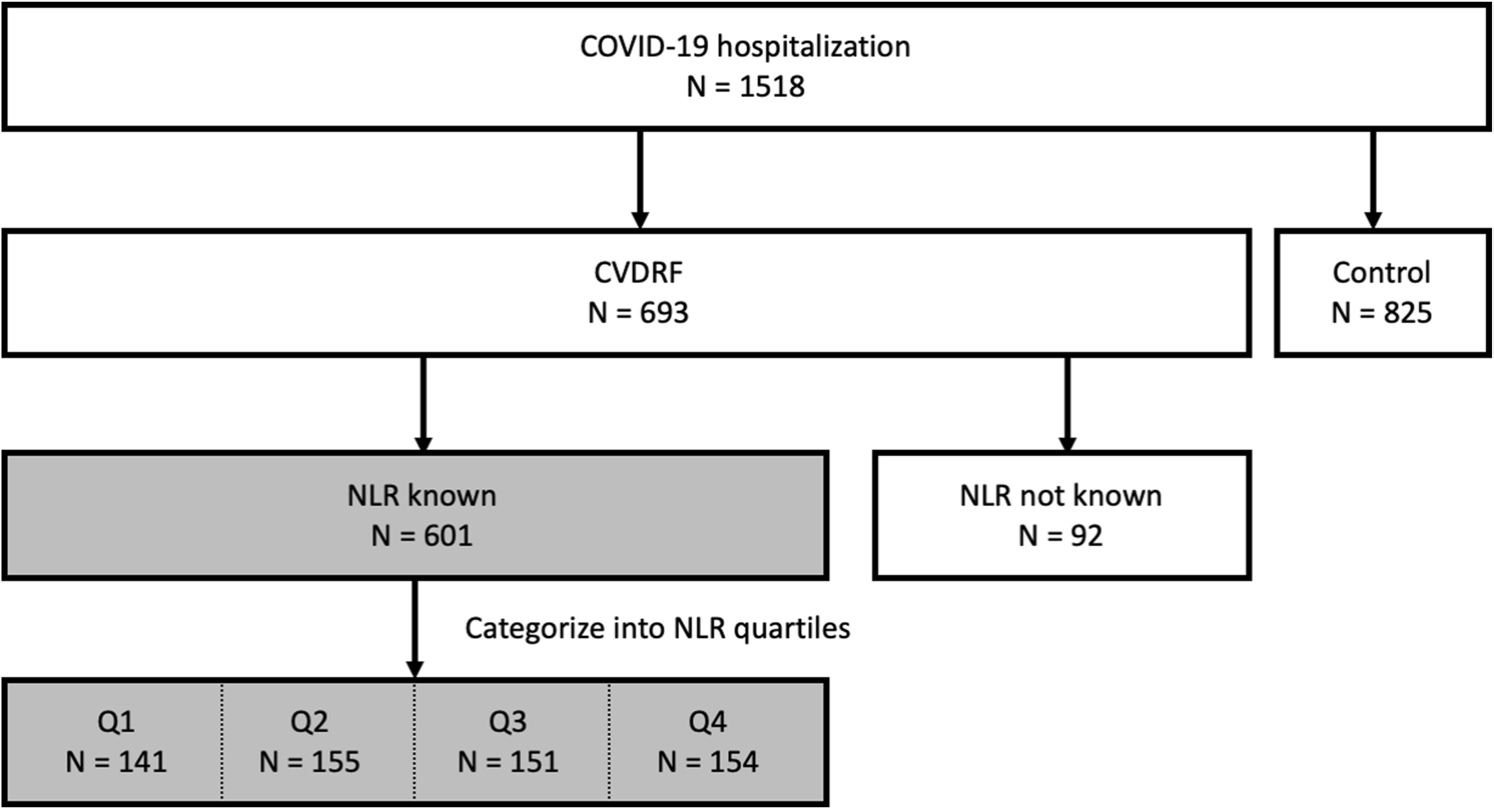
Study population. Patients included in the analysis are in the shaded boxes, N = 601. Q1–Q4 correspond to quartiles of the baseline NLR.

A list of all studied and excluded variables is made available through a Mendeley data repository (available at https://doi.org/10.17632/66djg6mmzf.2). Patients were divided into four groups according to the quartiles of baseline NLR values as previously reported^13,14^, to outline the characteristics of the cohort as the first step of the analysis. Patients whose NLR data were not available were excluded from the analysis. A schematic of the study population is shown in Fig. 1. The contribution of NLR to in-hospital mortality was analyzed as the primary endpoint using the statistical methods described below.

### Conventional statistical analysis

Data are shown as percentage for categorical variables and median (interquartile ranges, IQR) for continuous variables. One-way ANOVA and Kruskal–Wallis tests for continuous variables and χ^2^ tests for qualitative variables were used for between-group comparisons based on data distribution. Kaplan–Meier survival analysis was performed to compare 30-day in-hospital mortality among individuals in each NLR quartile. Multivariable Cox proportional hazards models were used to determine the hazard ratios and 95% CI for each factor. The validity of the proportional hazard assumption was verified using scaled Schoenfeld residuals. Receiver operating characteristic (ROC) curves were generated to obtain the area under the curve (AUC) as a predictor of mortality from the NLR values. The optimal cut-off value for predicting 30-day mortality was determined based on the Youden index.

Statistical significance was defined as a P-value of < 0.05. All statistical analyses were performed using SciPy, a Python library, and SPSS statistical package (Version 12, SPSS Inc, Chicago IL, USA).

### Clustering analysis and data visualization

We used t-stochastic neighborhood embedding (t-SNE)^17^ to visualize patients’ clinical characteristics. Scikit-learn, a Python machine learning library, was used for the analysis. All demographic and clinical variables at baseline and follow-up were included in the analysis, except for those that were missing in > 30% of patients. In the case of missing values, data were replaced with the item mean as previously described^18^. After confirming that the major clusters were consistently identified through different values of perplexity and iteration numbers (Supplementary Fig. S1), the default parameters of Scikit-learn (dimension = 2, perplexity = 30, learning rate = 200, and iteration number = 1000) were used to create a 2D map. Parameters of interest (mortality, intubation, baseline NLR, and follow-up NLR) were displayed on each data point as a heat map.

## Results

### Baseline characteristics and in-hospital outcomes of COVID-19 patients according to the quartiles of NLR

The curated dataset included 601 patients with 260 variables. The median number of days from symptom onset to hospital admission was seven (IQR 3–10). The median age of the cohort was 70 (IQR 58–80) years, and 65.4% of the participants were male. Further, 23.1% of the patients (n = 139) required mechanical ventilation and the overall number of in-hospital deaths was 98 (16.3%). Table 1 compares the baseline characteristics and in-hospital outcomes of COVID-19 patients according to the quartiles of NLR (Q1 0–2.57 n = 141, Q2 2.58–4.44 n = 155, Q3 4.45–7.48 n = 151, and Q4 7.49–84 n = 154). The median age was significantly different between the groups (p = 0.013). The higher quartile included a significantly higher number of male patients (p = 0.003). Significant differences were observed in the prevalence of diabetes mellitus and previous myocardial infarction (p = 0.008 and p = 0.028, respectively). Shortness of breath and dyspnea were more prevalent in the group of patients with higher NLR values (Q1 22.7%, Q2 25.8%, Q3 39.7%; Q4, 46.1%; p < 0.001). Antiviral drugs and steroids were used more frequently in patients with a high baseline NLR. Regarding clinical outcomes, the length of hospital stay (days) was significantly longer in patients with a higher baseline NLR (Q1 15 (10–22), Q2 17 (11–27.5) Q3 18 (12–28), and Q4 20 (12–31.5); p < 0.013). Mechanical ventilation with intubation was significantly frequent among the higher NLR quartiles (Q1 6.4%, Q2 16.1%, Q3 27.8%, and Q4 40.9%; p < 0.001). Hospital mortality significantly increased with an increase in the baseline NLR quartile (Q1 6.3%, Q2 11.0%, Q3 20.5%, and Q4 26.6%; p < 0.001).

**Table 1:** Demographics and clinical characteristics of COVID-19 patients according to baseline NLR quartiles.

Variable	Overall (0–84) N = 601	Q1 (0–2.57) N = 141	Q2 (2.58–4.44) N = 155	Q3 (4.45–7.48) N = 151	Q4 (7.49–84) N = 154	P-value
**Demographic**						
Age	70 (58–80)	63 (53–80)	71 (57–79)	70 (60–79)	72 (61–80)	0.013
Male n (%)	393 (65.4)	76 (53.9)	101 (65.1)	101 (66.9)	115 (74.7)	0.003
BMI (kg/m^2^)	23.8 (20.9–26.9)	24.2 (22.4–27.4)	23.9 (21.0–26.9)	23.0 (20.1–26.8)	22.8 (20.6–26.47)	0.218
**Comorbidity**						
Hypertension n (%)	445 (74.0)	106 (75.1)	112 (72.2)	118 (78.1)	109 (70.8)	0.470
Diabetes mellitus n (%)	230 (38.3)	38 (26.9)	59 (38.1)	63 (41.7)	70 (45.4)	0.008
Dyslipidemia n (%)	233 (38.7)	49 (34.7)	72 (46.4)	56 (37.1)	56 (36.3)	0.146
Heart failure n (%)	56 (9.3)	8 (5.6)	16 (10.3)	14 (9.2)	18 (11.6)	0.330
CHD n (%)	64 (10.6)	15 (10.6)	15 (9.6)	15 (9.9)	19 (12.3)	0.874
OMI n (%)	27 (4.5)	3 (2.1)	3 (1.9)	12 (7.9)	9 (5.8)	0.028
AF n (%)	56 (9.3)	10 (7.0)	16 (10.3)	14 (9.2)	16 (10.4)	0.748
COPD n (%)	33 (5.5)	5 (3.5)	8 (5.2)	8 (5.3)	12 (7.8)	0.450
CKD n (%)	45 (7.5)	4 (2.8)	12 (7.7)	16 (10.6)	13 (8.4)	0.081
**Symptom**						
Maximum body temperature (Celsius)	38.0 (37.6–38.6)	38.0 (37.2–38.5)	38.0 (37.6–38.6)	38.0 (37.6–38.6)	38.2 (37.8–39.0)	0.002
Cough n (%)	292 (48.6)	67 (47.5)	77 (49.6)	70 (46.3)	78 (42.7)	0.872
Sputum production n (%)	113 (18.8)	27 (19.1)	31 (20.0)	26 (17.2)	29 (18.8)	0.939
Sore throat n (%)	64 (10.6)	19 (13.4)	16 (10.3)	14 (9.2)	15 (9.7)	0.651
Nasal obstruction n (%)	27 (4.5)	8 (5.6)	8 (5.1)	6 (4.0)	5 (3.2)	0.735
Myalgia n (%)	9 (0.5)	2 (1.4)	1 (0.6)	2 (1.3)	4 (2.6)	0.559
Fatigue n (%)	203 (33.8)	43 (30.5)	50 (32.2)	56 (37.1)	54 (35.1)	0.638
Gastrointestinal symptoms n (%)	85 (14.1)	15 (10.6)	22 (14.2)	27 (17.9)	21 (13.6)	0.362
Headache n (%)	49 (8.1)	15 (10.6)	10 (6.4)	12 (7.9)	12 (7.8)	0.615
Shortness of breath / Dyspnea n (%)	203 (33.8)	32 (22.7)	40 (25.8)	60 (39.7)	71 (46.1)	< 0.001
**Treatment**						
Antiviral drug n (%)	329 (54.7)	38 (27.0)	84 (54.2)	96 (63.6)	111 (72.1)	< 0.001
Ciclesonide n (%)	154 (25.7)	27 (19.1)	34 (22.1)	38 (25.2)	55 (35.7)	0.006
Hydroxychloroquine n (%)	35 (5.8)	6 (4.2)	9 (5.8)	8 (5.3)	12 (7.8)	0.615
Tocilizumab n (%)	17 (2.8)	1 (0.7)	3 (1.9)	6 (4.0)	7 (4.5)	0.164
Steroids n (%)	228 (38)	31 (22.0)	54 (35.1)	50 (33.1)	93 (60.4)	< 0.001
**Outcome**						
Days of hospitalization	17 (11–27)	15 (10–22)	17 (11–27.5)	18 (12–28)	20 (12–31.5)	0.013
The need for mechanical ventilation n (%)	139 (23.1)	9 (6.4)	25 (16.1)	42 (27.8)	63 (40.9)	< 0.001
In-hospital death n (%)	98 (16.3)	9 (6.3)	17 (11.0)	31 (20.5)	41 (26.6)	< 0.001

Data are shown as percent for categorical and as median (IQR) for continuous variables.

*CHD* coronary heart disease, *OMI* old myocardial infarction, *AF* atrial fibrillation, *BMI* body mass index, *COPD* chronic obstructive pulmonary disease, *CKD* chronic kidney disease.

The median NLR at follow-up was 3.05 (IQR 1.95–5.77), which was significantly higher in the patients who showed high NLR at baseline. The median number of days from hospital admission to the last blood test (follow-up) was 13 (IQR 6–23), while the median number of days of hospitalization was 17 (IQR 11–27).

### Baseline laboratory parameters of COVID-19 patients according to baseline NLR quartiles

Laboratory data at admission were also compared among the baseline NLR quartiles. As shown in Table 2, the absolute white blood cell count was significantly higher in the higher NLR quartile groups (Q1 4400 (3700–5800), Q2 5100 (4150–6400), Q3 6100 (4950–7430), and Q4 8300 (6125–11,122); p < 0.001). The median NLR at follow-up was 3.05 (IQR 1.95–5.77), which was significantly higher in the patients who showed high NLR at baseline. The hemoglobin levels were significantly different among the groups. NLR at follow-up was higher in the group with high baseline NLR. As for other parameters, significant differences were observed in D-dimer, CRP, and LDH levels.

**Table 2:** Laboratory parameters of COVID-19 patients according to baseline NLR quartiles.

Variable	Overall (0–84) N = 601	Q1 (0–2.57) N = 141	Q2 (2.58–4.44) N = 155	Q3 (4.45–7.48) N = 151	Q4 (7.49–84) N = 154	P-value
**Blood count**						
WBC (counts/μL)	5700 (4370–7500)	4400 (3700–5800)	5100 (4150–6400)	6100 (4950–7430)	8300 (6125–11,122)	< 0.001
Neutrophil fraction	75.0 (64.6–82.6)	57.9 (54.0–61.4)	70.3(66.2–73.0)	79.0 (76.3–81.1)	87.3 (84.0–90.4)	< 0.001
Lymphocyte fraction	16.5 (10.7–25.0)	31.3 (28.0–35.6)	20.1 (18.2–22.7)	14.0 (12.2–15.5)	7.9 (5.5–9.5)	< 0.001
Hemoglobin (g/dL)	13.4 (11.6–14.7)	13.6 (12.3–14.9)	13.5 (11.5–14.9)	13.5 (11.4–14.8)	12.7 (11.4–14.4)	0.007
Platelet (× 10^4^ counts/μL)	20.7 (19.9–21.4)	19.9 (18.8–21.1)	20.8 (19.4–22.3)	20.8 (19.2–22.3)	21.0 (19.3–22.7)	0.760
NLR at follow up	3.05 (1.93–5.77)	1.88 (1.37–2.79)	3.04 (2.00–4.56)	3.50 (2.46–6.42)	4.28 (2.33–9.53)	< 0.001
**Coagulation**						
D-dimer (μg/mL)	1.49 (0.80–2.80)	0.95 (0.60–1.60)	1.20 (0.70–2.22)	1.60 (0.91–3.22)	2.25 (1.33–6.12)	< 0.001
PT (s)	13.8 (11.9–85.0)	12.4 (11.6–95.7)	13.1 (11.7–85.5)	13.3 (12.1–83.0)	18.7 (12.6–83.0)	0.471
APTT (s)	33.9 (30.0–38.3)	32.1 (29.5–35.0)	34.3 (29.8–38.2)	34.1 (31.4–38.6)	34.1 (30.0–39.4)	0.443
**Others**						
CRP (mg/dL)	5.5 (1.5–11.4)	1.2 (0.2–3.8)	3.7 (1.1–7.7)	7.1 (3.8–12.3)	12.2 (7.5–17.0)	< 0.001
LDH (IU/L)	288 (223–411)	230 (194–273)	255 (210–346)	340 (254–456)	396 (281–498)	< 0.001
Cre (mg/dL)	0.82 (0.66–1.05)	0.79 (0.61–0.92)	0.82 (0.64–1.02)	0.81 (0.67–1.10)	0.90 (0.70–1.19)	0.058

Data are shown as percentage for categorical and as median (IQR) for continuous variables.

*PT* Prothrombin time, *APTT* activated partial thromboplastic time, *LDH* lactate dehydrogenase, *Cre* creatinine.

### Survival analysis according to NLR quartiles

**Figure 2:**
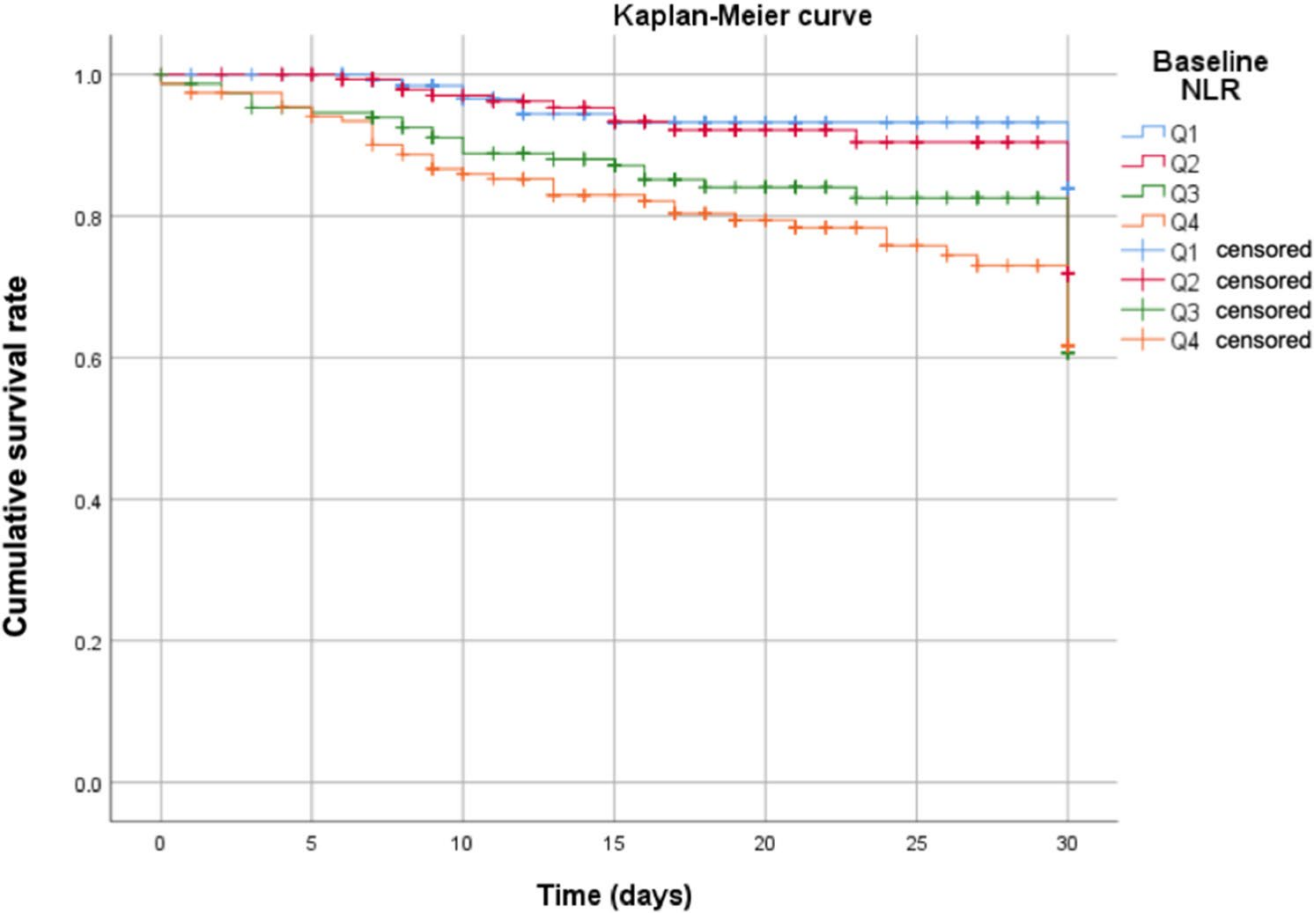
Kaplan–Meier curves for 30-day in-hospital survival among quartiles of baseline NLR. Cumulative probabilities of survival with increasing NLR values were shown. Q1–Q4 correspond to quartiles of the baseline NLR.

Kaplan–Meier curves show the different in-hospital mortality according to NLR quartiles, as shown in Fig. 2. Briefly, cumulative mortality increased as the quartile of baseline NLR increased. The paired log-rank test revealed significant differences in survival for Q1 vs Q3 (p = 0.017), Q1 vs. Q4 (p < 0.001), Q2 vs. Q3 (p = 0.034), and Q2 vs. Q4 (p < 0.001). There were no significant differences in survival between Q1 and Q2 (p = 0.706) or between Q3 and Q4 (p = 0.121). Multivariable Cox regression analysis revealed that older age, male sex, higher BMI, higher creatinine, and higher CRP values were significantly associated with 1-month mortality, while the baseline NLR was not (Table 3).

**Table 3:** Factors associated with 30-day mortality in hospitalized patients with COVID-19.

Covariates	Hazard ratio (95% CI)	P-value
Age	1.120 (1.092–1.149)	< 0.001
Male sex	1.893 (1.324–3.759)	0.005
BMI	1.093 (1.035–1.155)	0.001
Cre	1.197 (1.084–1.323)	< 0.001
CRP	1.086 (1.057–1.115)	< 0.001
Baseline NLR	1.014 (0.989–1.041)	0.274

*BMI* body mass index, *Cre* Creatinine, *CRP* C-reactive protein.

### Prognostic value of the baseline and follow-up NLR for disease fatality

**Figure 3:**
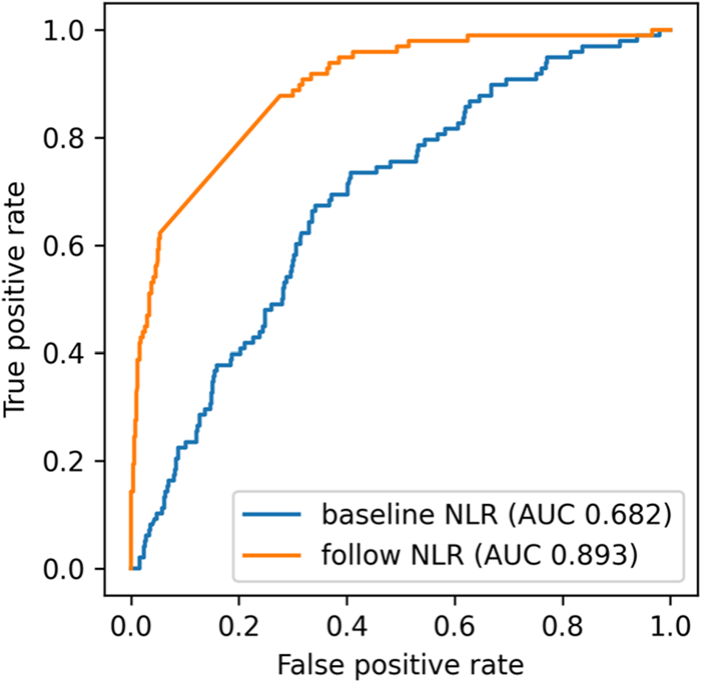
Receiver operating characteristics (ROC) for the prediction of mortality from NLR levels. ROC curves for mortality prediction were shown for baseline NLR (red) and follow-up NLR (blue) respectively. AUC, area under the curve.

As shown in Fig. 3, the AUC for predicting in-hospital death based on baseline NLR was only 0.682. In contrast, the AUC for predicting in-hospital death by the follow-up NLR was 0.893. The optimal cut-off value of the baseline NLR for predicting in-hospital death was 5.39.

### Data visualization using t-SNE

**Figure 4:**
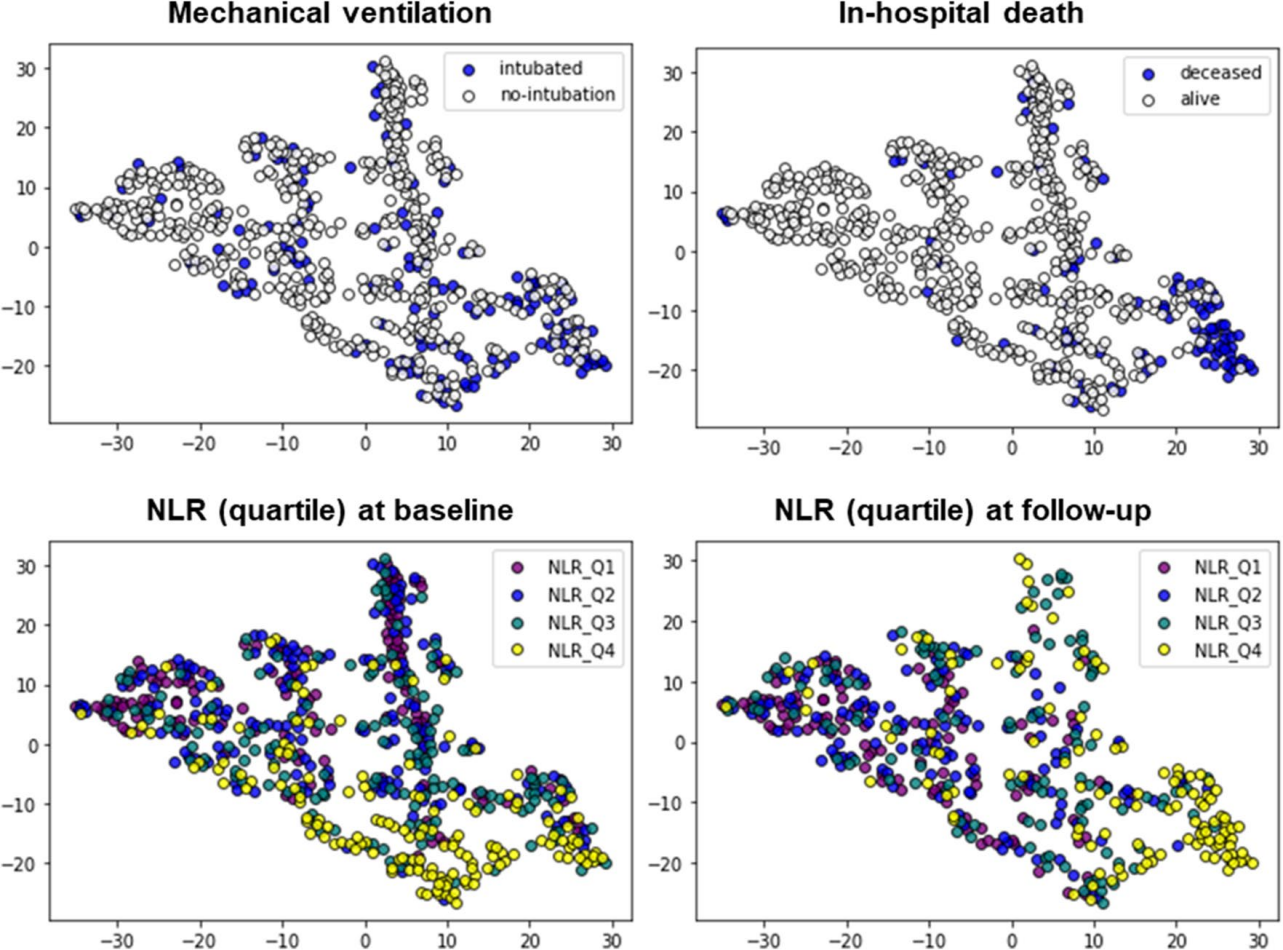
Two-dimensional mapping of study population by t-SNE. Axes represents arbitrary unit determined based on t-SNE. Target variables are indicated by color-bar in each mapping.

Based on the 2D mapping shown in Fig. 4, we identified two distinct clusters in the fourth quadrant (lower right), one of which was characterized by high in-hospital mortality. The population that required mechanical ventilation was also seen in the fourth quadrant and seemed to overlap with the population with a high baseline NLR. Patients who survived in the fourth quadrant had a lower NLR at the follow-up.

## Discussion

To date, several studies have reported that severe systemic inflammation is associated with higher incidence of CVD^12,19–22^. The results of the current study illustrate the clinical implications of the NLR in COVID-19 patients with CVDRF. The main findings were that in-hospital mortality was higher among patients with higher baseline NLR, but the predictive performance of baseline NLR for fatality was insufficient. Similarly, survival analysis showed that baseline NLR was not significantly associated with 30-day mortality, unlike other parameters, including age and sex. In contrast, NLR at follow-up was significantly higher in deceased patients than in those who survived, leading to a high area under the ROC curve for the prediction of mortality. Further analysis using unsupervised machine learning-based visual mapping revealed that a cluster with a high baseline NLR tended to require mechanical ventilation but did not necessarily show high mortality.

Previous reports have revealed an association between NLR and disease severity or mortality in COVID-19 patients. However, the cut-off values for prognosis prediction vary across studies, probably due to the heterogeneity of the cases studied^23–26^. In addition, the cut-off value calculated in this study (baseline NLR > 5.39) was higher than any of the previously reported values. According to a study by Caillon et al., NLR was not selected as an important variable in their mortality prediction model^26,27^. A possible factor for the varying results could be the timing of data collection. A recent study by Jimeno et al. showed that the peak NLR value and the rate of NLR increase, but not the NLR value at hospital admission, are significantly associated with mortality in COVID-19 patients^28^. Although the prevalence of comorbidities has not been reported in detail, their findings are consistent with our results that later data are more reflective of disease severity. Interestingly, the median number of days from symptom onset to hospital admission in the data of Jimeno et al. was the same as ours, with a median of 7 days. During the study period, the Japanese government mandated the hospitalization of all patients with COVID-19 regardless of disease severity during patient enrollment^29^. Therefore, laboratory data at the time of hospitalization may have been collected before the onset of the disease or at a relatively mild stage compared with in reports from other countries. In any case, it should be noted that the follow-up blood tests in our study were collected at the end of hospitalization; therefore, they are not clinically useful in predicting disease severity.

Another unique aspect of our research lies in our application of the cluster analysis method using unsupervised machine learning (specifically, t-SNE). Analysis using machine learning has become a trend in the field of cardiovascular medicine owing to its advantages over existing statistical methods^30^. A nonlinear dimensionality reduction technique, t-SNE is a manifold learning algorithm that is commonly used for the visualization of high-dimensional data in genomic analysis. The use of this algorithm is not limited to genomic data but also includes the analysis of electronic medical record data and posturography data in neurodegenerative diseases^31,32^. Recently, De Canniere et al. reported the efficacy of two-dimensional (2D) visualization of 6-min walking test data using t-SNE-based mapping^33^. The 2D map allows for the simultaneous assessment of the relative similarity of all subjects in our dataset, along with the distribution of their clinical characteristics. In this study, we visualized the different distributions of NLR quartiles according to the time at which the data were obtained (admission or follow-up). We believe that this method is useful for phenotyping patient groups, as it can represent high-dimensional data in a human-interpretable (2D) way.

In summary, we have shown that while NLR clearly reflects disease activity, it does not necessarily predict future disease severity based on values at admission. Further validation with therapeutic intervention is required to confirm the usefulness of the NLR as a biomarker.

## Limitations

The current study has several limitations. First, this was a retrospective study, and there were considerable missing values for baseline serum biomarkers, especially cTn and BNP/NT-proBNP. The missing data might result in our univariate and multivariate analyses of cardiac biomarkers differing from the results of previous studies. In addition, patients with mild disease had only one blood test performed during hospitalization, which may have led to bias due to missing data. The second limitation was the study population. As mentioned earlier, this study used a registry that enrolled patients with relatively mild disease, which may not necessarily reflect the current status of most hospitalized patients. Recently, the proportion of new variant strains of SARS-CoV-2 has increased in hospitalized patients, and the variant strains have been reported to differ from conventional strains in infectivity and severity of disease^34,35^. Therefore, the influence of the variant strains is a major limitation that cannot be addressed in this study. Third, we were unable to show the clear benefit of using the ratio of neutrophils to lymphocytes, rather than using their sole values. As shown in Supplementary Fig. S2, the advantage of using NLR only emerged at the time of follow-up. Fourth, when we excluded CRP from the covariates in the multivariable Cox proportional hazards regression model, the baseline NLR became an independent factor for predicting 1-month mortality (Supplementary Table S1). Therefore, multicollinearity was suspected between CRP level and NLR. Lastly, the CLAVIS-COVID registry analyzed in this study focuses on patients with CVDRF, and no blood test data are available for the control group (subjects without CVDRF). Therefore, it is unclear whether patients with CVDRF exhibit a higher NLR than those without.

## Conclusion

NLR values may have prognostic implications among hospitalized COVID-19 patients with CVDRF; however, their significance depends on the timing of data collection. In predicting the prognosis of COVID-19 patients with CVDRF using the NLR, one should focus on changes in values over time.

## Supplementary Information

Supplementary Information.
