# Supplementary material for: Predictive value of neutrophil-to-lymphocyte ratio for the fatality of COVID-19 patients complicated with cardiovascular diseases and/or risk factors
Source: Sci Rep. 2022 Aug 10;12:13606. doi: 10.1038/s41598-022-17567-4 (PMC9364304; doi:10.1038/s41598-022-17567-4)
Supplement: Supplementary file 1 — Supplementary Information. [file 41598_2022_17567_MOESM1_ESM.docx]

**Supplementary data**


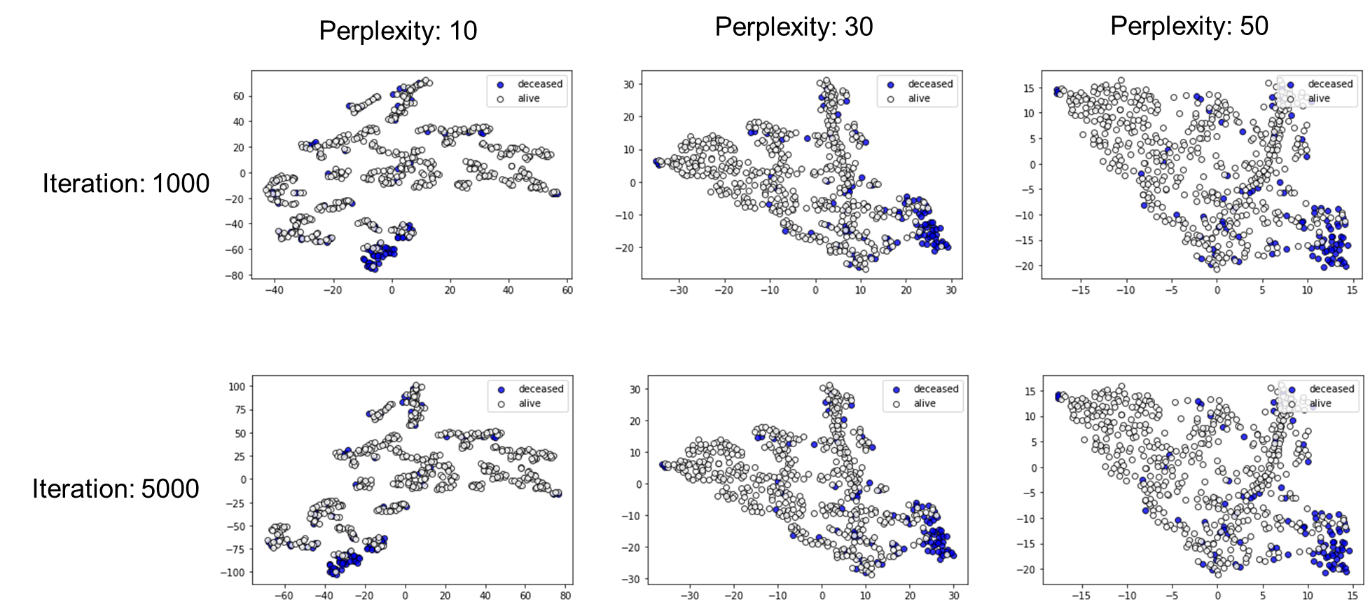


**Supplementary Figure S1. Outcome of t-SNE with different parameters.** T-SNE mapping was conducted under a variety of parameters under fixed random state. Colored data points indicate ceased subjects. The cluster with high in-hospital mortality was consistent through different perplexities.


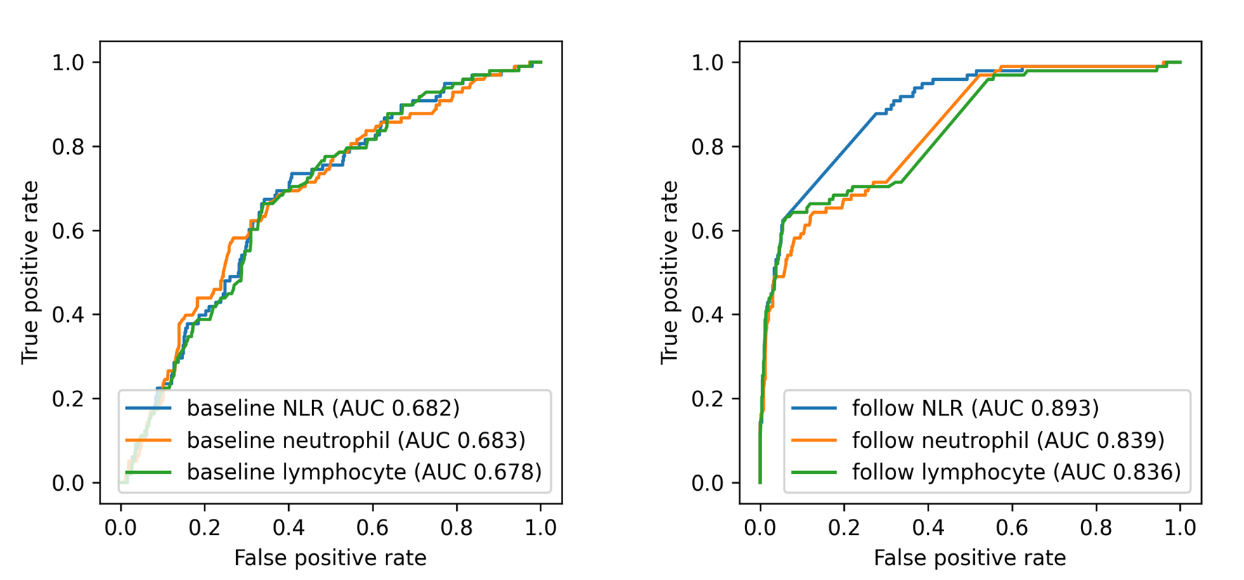


**Supplementary Figure S2. The predictive performance of neutrophil, lymphocyte and NLR for COVID-19 mortalit**y. The AUC of baseline NLR was comparable to neutrophil or lymphocyte alone (left panel). The advantage of taking ratio of neutrophil to lymphocyte emerges at the time of follow-up (right panel). NLR, neutrophil-to-lymphocyte ratio; AUC, area under the curve.

| Covariates | Hazard ratio (95% CI) | P-value |
| --- | --- | --- |
| Age | 1.084 (1.084-1.148) | <0.001 |
| Male sex | 2.964 (1.560-5.631) | 0.001 |
| BMI | 1.107 (1.042-1.177) | 0.001 |
| Cre | 1.126 (0.966-1.313) | 0.129 |
| Baseline NLR | 1.025 (1.003-1.048) | 0.024 |

**Supplementary Table S1. Results of multivariable Cox proportional hazards regression model without using CRP as a covaritate.** Body mass index. BMI; Creatinine, Cre.
